# Limitations in metabolic plasticity after traumatic injury are only moderately exacerbated by physical activity restriction

**DOI:** 10.1038/s44324-024-00006-5

**Published:** 2024-04-06

**Authors:** Angela S. Bruzina, Christiana J. Raymond-Pope, Kevin J. Murray, Thomas J. Lillquist, Katelyn M. Castelli, Shefali R. Bijwadia, Jarrod A. Call, Sarah M. Greising

**Affiliations:** 1 School of Kinesiology, University of Minnesota, Minneapolis, MN 55455 USA; 2 Center for Metabolomics and Proteomics, University of Minnesota, Minneapolis, MN 55455 USA; 3 Department of Physiology and Pharmacology, University of Georgia, Athens, GA 30602 USA; 4 Regenerative Bioscience Center, University of Georgia, Athens, GA 30602 USA

**Keywords:** Physiology, Metabolic diseases, Metabolomics, Metabolic disorders

## Abstract

Following traumatic musculoskeletal injuries, prolonged bedrest and loss of physical activity may limit muscle plasticity and drive metabolic dysfunction. One specific injury, volumetric muscle loss (VML), results in frank loss of muscle and is characterized by whole-body and cellular metabolic dysfunction. However, how VML and restricted physical activity limit plasticity of the whole-body, cellular, and metabolomic environment of the remaining uninjured muscle remains unclear. Adult mice were randomized to posterior hindlimb compartment VML or were age-matched injury naïve controls, then randomized to standard or restricted activity cages for 8-wks. Activity restriction in naïve mice resulted in ~5% greater respiratory exchange ratio (RER); combined with VML, carbohydrate oxidation was ~23% greater than VML alone, but lipid oxidation was largely unchanged. Activity restriction combined with VML increased whole-body carbohydrate usage. Together there was a greater pACC:ACC ratio in the muscle remaining, which may contribute to decreased fatty acid synthesis. Further, β-HAD activity normalized to mitochondrial content was decreased following VML, suggesting a diminished capacity to oxidize fatty acids. The muscle metabolome was not altered by the restriction of physical activity. The combination of VML and activity restriction resulted in similar ( ~ 91%) up- and down-regulated metabolites and/or ratios, suggesting that VML injury alone is regulating changes in the metabolome. Data supports possible VML-induced alterations in fatty acid metabolism are exacerbated by activity restriction. Collectively, this work adds to the sequalae of VML injury, exhausting the ability of the muscle remaining to oxidize fatty acids resulting in a possible accumulation of triglycerides.

## Introduction

Regular physical activity elicits positive adaptations in skeletal muscle function and whole-body metabolism, improving the body’s ability to readily adapt to changes in metabolic demand, a concept known as metabolic flexibility^1,2^. In contrast, physical inactivity (i.e., sedentarism), such as in the case of bedrest following injury or in disease conditions, leads to skeletal muscle atrophy, muscle weakness, and impaired metabolic flexibility. Volumetric muscle loss (VML)^3^, can be associated with prolonged periods of bedrest and/or inactivity; however, the extent to which inactivity influences the injury sequalae in the remaining muscle, negatively or positivity, after a VML injury is unclear. Addressing this knowledge gap can advance our understanding of metabolic risk factors associated with VML injuries (e.g., obesity, type II diabetes) and aid in the development of interventions to support long-term health of the patient.

The sequalae of VML injury prohibits the long-term recovery of skeletal muscle function^3–5^. The irrecoverable loss of muscle function is due to a combination of factors, including loss of contractile tissue and capacity for endogenous skeletal muscle regeneration, chronic inflammation^6–8^ and fibrotic tissue accumulation^9–11^, primary and secondary denervation^12,13^, and metabolic dysfunction^14–19^. In particular, the metabolic impact of VML is evident at both the whole-body and local muscle levels, despite no change in ambulation following injury. Whole-body evaluations indicate injury decreases metabolic flexibility chronically, corresponding with decreased metabolic rate and respiratory exchange ratio (RER)^18^. There are also alterations in the distribution of slow and fast expressing muscle fibers, and dysregulated mitochondrial function following VML^18,20^, the latter of which is further exacerbated by a high-sugar, high-fat Western diet^21^. When physical activity is restricted following VML, RER increases which is reflected by a lower lipid oxidation, mirroring clinical conditions of bedrest. Interestingly, physical activity restriction does not hinder muscle function beyond the dysfunction imparted by the injury itself^16,17^. Although investigations have noted physical activity restriction following VML alters whole-body and local muscle metabolism^17^, it is unknown how these metabolic observations following VML compare to a healthy uninjured control group undergoing physical activity restriction for the same length of time.

Physical activity and inactivity are known to cause acute and chronic changes to the metabolome by altering the composition and concentration of metabolic intermediates within skeletal muscle. These metabolic intermediates, metabolites, are implicated in various musculoskeletal pathologies, including metabolic inflexibility, mitochondrial dysfunction, and impaired muscle regeneration^22,23^. Reduced physical activity is associated with changes in lipid-related metabolites, such as accumulation of long-chain fatty acids and acylcarnitine^24^. Physical activity can produce acute changes in skeletal muscle metabolites, for example those related to enhanced β-oxidation, in which carnitine and acylcarnitine metabolites are released to buffer fatty acid oxidation flux^25^. However, if there are imbalances between fatty acid uptake and availability, such as those that can occur during reduced physical activity, there can be an excess of intracellular lipid depots and uncontrolled mitochondrial fatty acid influx^26^. Physical activity is known to induce positive whole-body adaptations alongside metabolomic changes, including enhanced whole-body lipid oxidation partly driven by increased insulin sensitivity^27^. In contrast, chronically reduced physical activity, particularly following traumatic musculoskeletal injury, results in chronic lipid oxidation dysfunction and alterations in polyamine metabolites^28^, which progressively worsens in injuries such as VML^17^. It remains unclear if the metabolome of the affected muscle is impacted with persistently reduced physical activity following musculoskeletal injury, specifically VML.

The objective of this work was to elucidate the impact of VML and reduced physical activity on the whole-body, cellular, and metabolomic environment of the remaining uninjured skeletal muscle. We hypothesized that combined restricted physical activity and VML injury would worsen function of the muscle remaining and negatively impact whole-body metabolism. Clinically and translationally, the combination of injury and reductions in physical activity can be common in both the short- and long-term management of VML injured limbs. Using complementary physiologic and metabolomic approaches, there was evidence for metabolite accumulation in the muscle remaining both following VML and VML injury combined with restricted activity, in particular metabolites related to triglycerides. Thus, mechanistic insights into fat oxidation in the muscle remaining were also evaluated.

## Results

### Animals and experimental design

At ~13 weeks of age, mice underwent a full thickness, unilateral VML injury to the posterior compartment muscle group of the hindlimb or served as age-matched injury naïve controls. All mice underwent and recovered without complications from surgery. Immediately following surgery or study start (naïve), mice were randomly assigned to standard (28 x 18 x 12.5 cm) or restricted cages (12.5 × 8.5 × 6.3 cm), which have been shown to reduce physical activity by ~50% and alter whole-body metabolism within the first week following restriction, compared to standard cages^16,17^. Mice underwent whole-body metabolic and physical activity measurements and glucose tolerance tests at 6- and 7-weeks post, respectively. Then at 8 weeks post ( ~ 21 weeks of age) in vivo muscle function was assessed.

Terminally at ~21 weeks of age, mice with restricted activity had the lowest body mass, independent of injury (main effect activity *p* = 0.026; Table 1). However, VML-injured mice, independent of physical activity status, had a substantially greater change in body mass ( ~ 11 g total) than injury naïve mice across 8 weeks (main effect injury *p* < 0.0001; Table 1). As expected, VML-injured mice had a smaller gastrocnemius muscle mass, even when normalized by body mass, compared with injury naïve (main effect injury *p* < 0.0001; Table 1).

**Table 1:** Animal weights and gastrocnemius muscle mass

	Unrestricted		Restricted		Two-way ANOVA *p*-value		
	Naïve	VML	Naïve	VML	Main Effect of Injury	Main Effect of Activity	Interaction
	(*n* = 8)	(*n* = 12)	(*n* = 8)	(*n* = 12)			
Pre-VML Body Mass (g)	29.0 ± 1.8	27.4 ± 2.3	28.0 ± 1.3	27.3 ± 2.3	0.101	0.433	0.494
Terminal Body Mass (g)	30.9 ± 1.8	30.5 ± 2.7	27.8 ± 1.6 †	30.1 ± 2.7 †	0.219	0.026	0.094
Change in Body Mass (%)	6.7 ± 3.1	11.6 ± 5.3 *	−0.7 ± 1.9	11.1 ± 7.8 *	< 0.0001	0.066	0.123
Left (injured) Gastrocnemius Mass (mg)	181.6 ± 10.5	139.7 ± 22.9 *	166.4 ± 10.2	138.6 ± 12.1 *	< 0.0001	0.116	0.171
Left (injured) Gastrocnemius Mass/Body Mass (mg/g)	5.89 ± 0.35	4.58 ± 0.62 *	6.18 ± 0.24	4.62 ± 0.20 *	< 0.0001	0.218	0.344

Data presented as mean ± SD. Significantly different than *Naïve; †Unrestricted.

### Skeletal muscle function

**Fig. 1:**
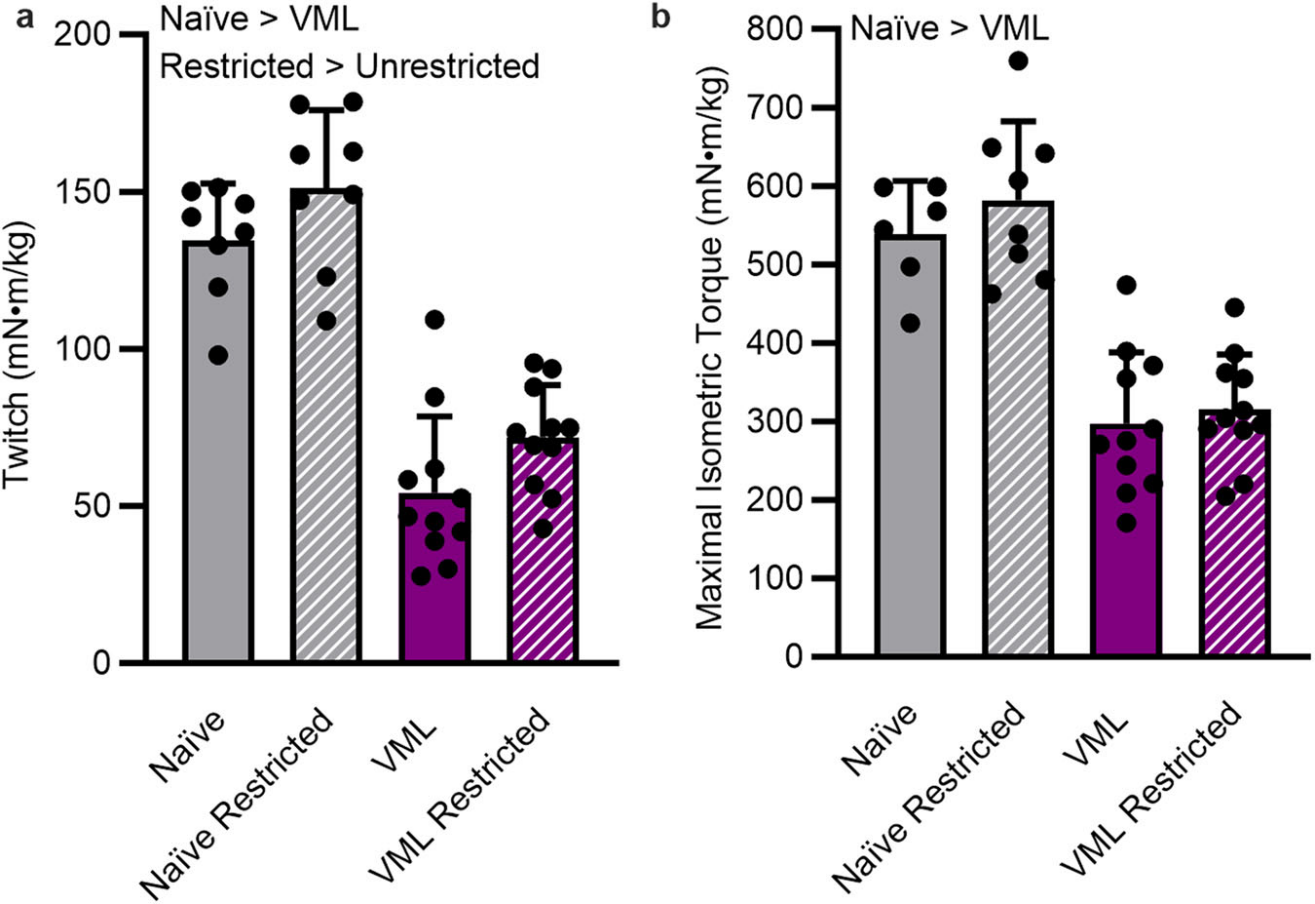
Terminal muscle function of the posterior compartment of the hindlimb. **a** A single in vivo muscle twitch was significantly impaired 8 weeks following VML injury, with physical activity restriction and VML injury (main effect injury *p* < 0.0001; activity *p* = 0.034; interaction *p* = 0.895). **b** VML injury resulted in a chronic deficit in maximal isometric torque that was not impacted by physical activity restriction (main effect injury *p* < 0.0001; activity *p* = 0.518; interaction *p* = 0.958). Individual data points represent a single mouse.

Terminally, VML-injured mice had significantly lower in vivo twitch and maximal isometric torque of the posterior muscle compartment normalized to body mass (main effect injury *p* < 0.0001; Fig. 1). With the restriction of physical activity, the twitch torque was greater (main effect activity *p* = 0.034; Fig. 1a), but there was no impact on maximal isometric torque (main effect injury *p* = 0.518; Fig. 1b). Maximal twitch and isometric torque tracings were evaluated for contractile properties, with all differences attributed to injury (main effect injury *p* ≤ 0.023; Table 2) but not the restriction of physical activity (main effect activity *p* ≥ 0.111; Table 2). Both the twitch half-relaxation time and time to peak twitch were lower following VML injury. Evaluation of the maximal isometric torque curve revealed significantly slower average rates of contraction and relaxation following injury, supporting chronic functional dysregulation of the injured muscle.

**Table 2:** In vivo contractile parameters

	Unrestricted		Restricted		Two-way ANOVA *p*-value		
	Naïve	VML	Naïve	VML	Main effect of injury	Main effect of activity	Interaction
	(*n* = 8)	(*n* = 12)	(*n* = 8)	(*n* = 12)			
Time to Peak Twitch (s)	0.028 ± 0.002	0.023 ± 0.003 *	0.027 ± 0.004	0.023 ± 0.005 *	0.001	0.872	0.703
½ Relaxation Time (s)	0.020 ± 0.001	0.015 ± 0.003 *	0.019 ± 0.004	0.015 ± 0.003 *	< 0.0001	0.663	0.561
Twitch : Tetanus	0.26 ± 0.05	0.18 ± 0.05 *	0.26 ± 0.04	0.23 ± 0.03 *	0.001	0.111	0.105
+dP/dt (mN·m/s)	235.3 ± 31.2	189.1 ± 70.9 *	239.1 ± 58.7	184.1 ± 64.2 *	0.023	0.977	0.838
−dP/dt (mN·m/s)	−340.6 ± 62.4	−194.7 ± 78.3 *	−333.5 ± 58.2	−211.6 ± 86.4 *	< 0.0001	0.851	0.645

Data represented as mean ± SD. Significantly different from *Naïve.

### Physical and whole-body metabolic activity

**Fig. 2:**
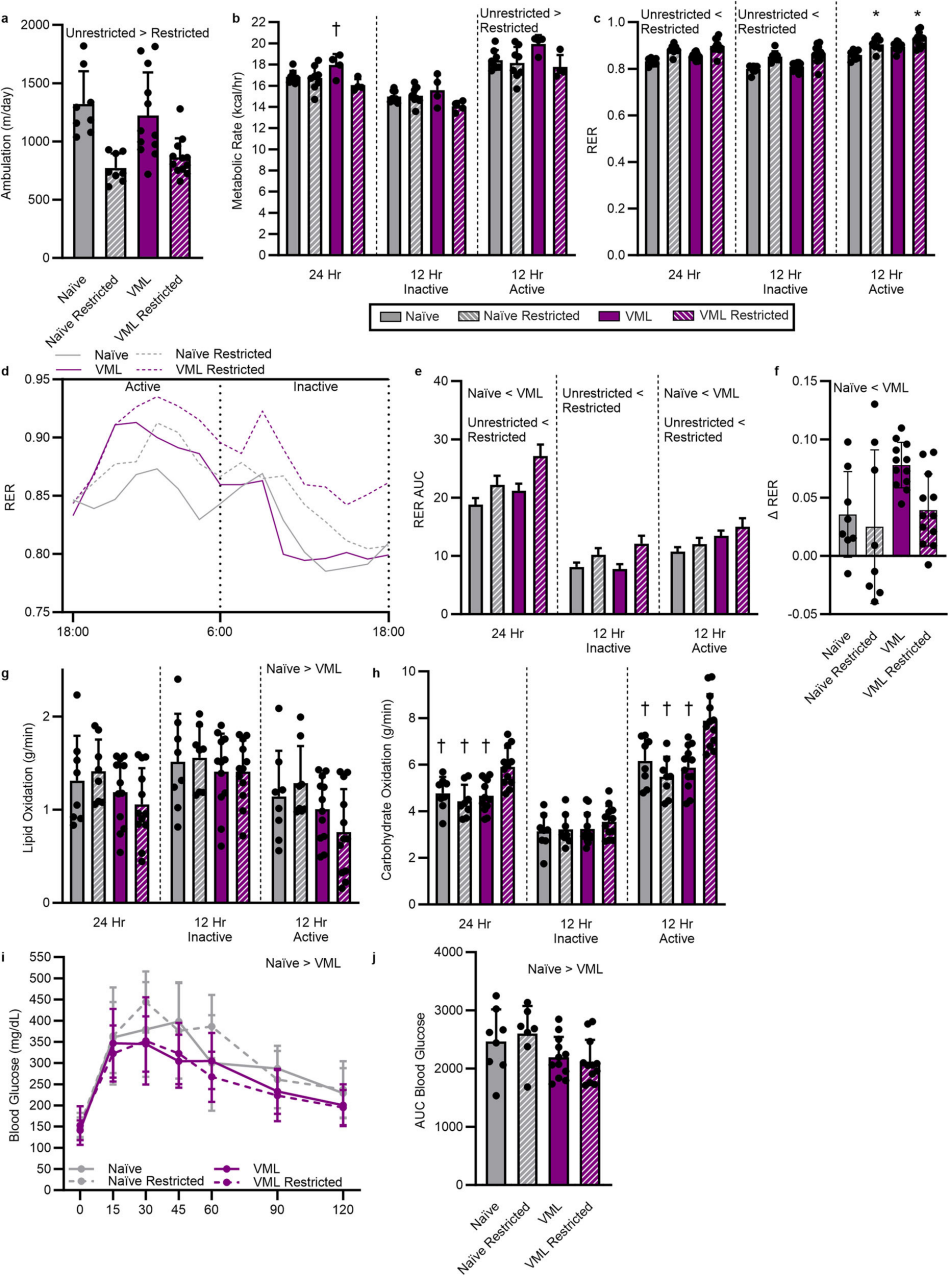
Evaluation of physical activity, whole-body metabolism, and glucose tolerance up to seven weeks following VML injury. **a** Total ambulation between groups (main effect injury *p* = 0.955); activity *p* < 0.0001; interaction *p* = 0.271. **b** Metabolic rate across 24-h (interaction *p* = 0.033); 12-h inactive (main effect injury *p* = 0.559; activity *p* = 0.001; interaction 0.419); 12-h active (main effect injury *p* = 0.278; activity *p* = 0.029; interaction *p* = 0.084). **c** RER across 24-h (main effect injury *p* = 0.187; activity *p* = 0.001; interaction *p* = 0.088); 12-h inactive (main effect injury *p* = 0.559; activity *p* = 0.001; interaction *p* = 0.419); 12-h active (interaction *p* = 0.047) **d** Fluctuations in RER over 24-h period **e**. RER AUC across 24-h (main effect injury *p* < 0.0001; activity *p* < 0.0001); 12-h inactive (main effect injury *p* = 0.426; activity *p* < 0.0001); 12-active (main effect injury *p* < 0.0001; activity *p* < 0.0001). **f** Quantified as the difference between the 12-h active and 12-h inactive period, Δ RER was greatest following VML injury (main effect injury *p* = 0.030; activity *p* = 0.058; interaction *p* = 0.269). **g** Lipid oxidation across 24-h (main effect injury *p* = 0.068; activity *p* = 0.894; interaction *p* = 0.365); 12-h inactive (main effect injury *p* = 0.334; activity *p* = 0.857; interaction *p* = 0.868); 12-h active (main effect injury *p* = 0.020; activity *p* = 0.707; interaction *p* = 0.157). **h** Carbohydrate oxidation across 24-h (interaction *p* = 0.001); 12-h inactive (main effect injury *p* = 0.295; activity *p* = 0.470; interaction *p* = 0.564); 12-h active (interaction *p* < 0.0001). **i** Glucose tolerance across time (main effect injury *p* < 0.0001; activity *p* = 0.597; time *p* < 0.0001). **j** Glucose tolerance AUC (main effect of injury *p* = 0.011; activity *p* = 0.826; interaction *p* = 0.462). Significantly different from *Naïve Unrestricted; †VML Restricted. Individual data points represent a single mouse.

At 6 weeks following VML injury, 24-h physical activity and whole-body metabolic activity were monitored. As expected, daily ambulation was ~40% less when activity was restricted (main effect activity *p* < 0.0001; Fig. 2a). Corresponding to reduced activity, VML-injured mice with restricted activity had a lower 24-h metabolic rate (interaction *p* = 0.032), due to reductions during the 12-h active period (main effect activity *p* = 0.029; Fig. 2b). In contrast, 24-h RER (main effect activity *p* = 0.001) was significantly higher, due to increased RER in both the 12-h inactive (main effect activity *p* = 0.001) and active periods (interaction *p* = 0.047), particularly following physical activity restriction (Fig. 2c). Activity restriction led to a greater 24-h RER, as calculated by AUC (main effect activity *p* < 0.0001; Fig. 2d–e). VML-injured mice with restricted physical activity exhibited the greatest RER AUC with a ~ 31% average increase across the 24-h period (*p* ≤ 0.0001). Injury resulted in a greater change in RER from inactive to active periods (i.e., Δ RER) with nearly a onefold increase over injury naïve (main effect injury *p* = 0.030; Fig. 2f). However, comparison of the VML injured restricted and non-restricted groups, revealed a blunted change in RER between active to inactive periods, suggesting metabolic inflexibility. The 24-h lipid oxidation was largely unaltered (*p* ≥ 0.068). Injured mice had lower lipid oxidation during the 12-h active period (main effect injury *p* = 0.020; Fig. 2g), but there were no differences in the inactive period (*p* ≥ 0.333). The collective changes in RER correspond to increased carbohydrate oxidation over the day (interaction *p* = 0.001) and during the 12-h active period (interaction *p* = 0.001), particularly for VML-injured mice (Fig. 2h). At 7 weeks following VML injury, fasting glucose tolerance was evaluated. Regardless of physical activity restriction, blood glucose levels were lower following VML-injury, suggesting improved glucose tolerance compared injury naïve (main effect injury *p* < 0.0001; Fig. 2i), blood glucose were also lower following VML when analyzed by AUC (main effect of injury *p* = 0.011; Fig. 2j).

### Muscle histologic quality

**Fig. 3:**
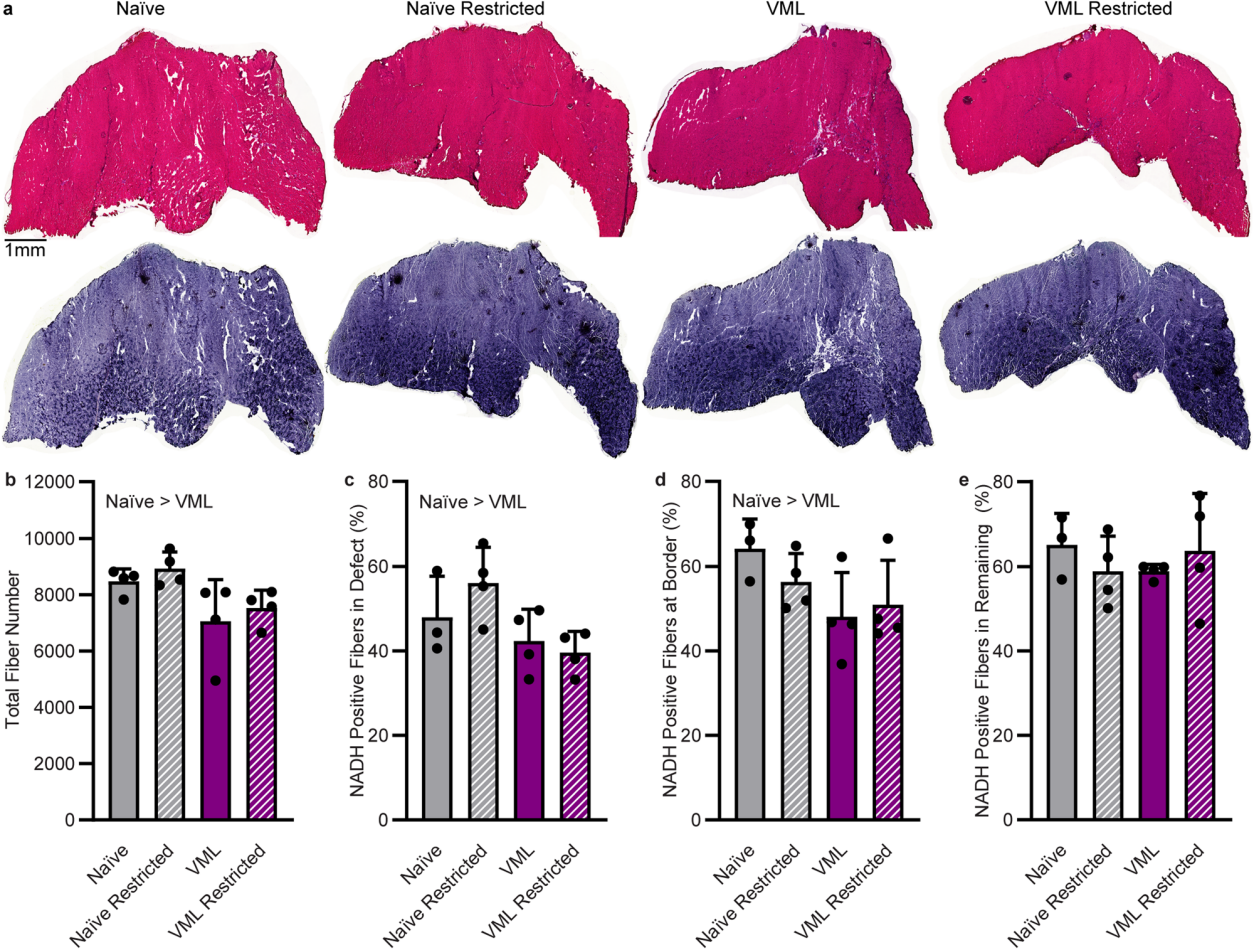
Histological evaluation of total muscle fiber number and oxidative properties of the muscle within and surrounding the defect and in the muscle remaining. **a** Representative serial images of the mid-belly of the gastrocnemius muscle to evaluate fiber number (H&E) and percentage NADH positive fibers, which was evaluated in a sub-set of muscles. Scale bas is 1 mm for all images. **b** Eight weeks following VML injury, total muscle fiber number was significantly lower than injury naïve (main effect injury *p* = 0.008; activity *p* = 0.316; interaction *p* = 0.978). **c** The percentage of NADH positive fibers within the defect region of the gastrocnemius muscle was lower following VML (main effect injury *p* = 0.018; activity *p* = 0.516; interaction *p* = 0.200). **d** This was similar to the injury border region (main effect injury *p* = 0.043; activity *p* = 0.607; interaction *p* = 0.277). **e** No differences in NADH expressing fibers in the remaining gastrocnemius muscle with injury or activity restriction (main effect injury *p* = 0.883; activity *p* = 0.889, interaction *p* = 0.259). Individual data points represent a single mouse.

The mid-belly of the gastrocnemius muscle was histologically evaluated within the VML defect and the remaining muscle. In line with the similarities in muscle function, the restriction of physical activity did not impact the total gastrocnemius muscle fiber number, with ~8700 fibers across the mid-belly of the muscle. The VML injury resulted in ~16% fewer total fibers across the muscle regardless of physical activity restriction (main effect *p* = 0.008; Fig. 3a, b). The oxidative capacity of the muscle was histologically evaluated by the positive expression of NADH within the fibers. Averaged across standardized evaluated regions, VML injury resulted in a lower percentage of NADH-positive fibers than injury naïve ( ~ 49% and 58%, respectively; main effect injury *p* = 0.005; activity *p* = 0.697; interaction *p* = 0.672). Suggesting an overall reduction in the oxidative capacity of fibers across the muscle with VML injury. Much of this reduction was in the standardized regions evaluated in the VML defect and along the border with the muscle remaining (main effect injury *p* ≤ 0.043; Fig. 3a, c, d). In the muscle remaining, there was no difference due to injury or physical activity across groups (*p* ≥ 0.259; Fig. 3a and e).

### Biochemical signaling of fatty acid and glucose metabolism

**Fig. 4:**
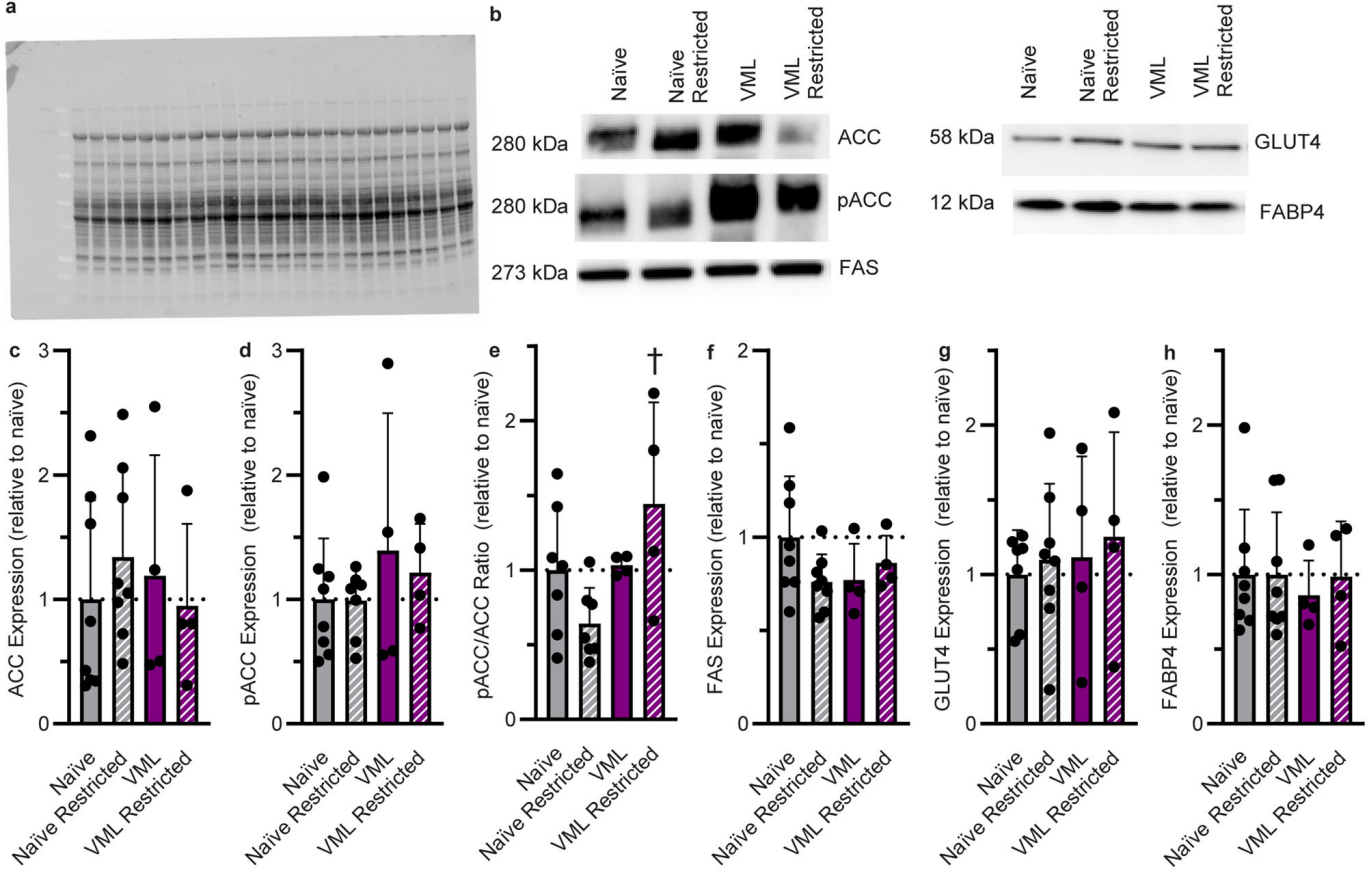
Terminal protein expression of fatty acid and glucose metabolism signaling in the gastrocnemius muscle and liver. Representative images of **a** a stain free blot for total lane protein quantification and **b** chemiluminescence blots for quantification of band intensity. **c** Lack of differences between ACC expression across all experimental groups (main effect injury *p* = 0.766; activity *p* = 0.885; interaction *p* = 0.395). **d** No significant difference of pACC expression across all experimental groups (main effect injury *p* = 0.216; activity *p* = 0.705; interaction *p* = 0.729). **e** VML restricted activity resulted in greater pACC ratio compared to other experimental groups (main effect injury *p* = 0.031; activity *p* = 0.885; interaction *p* = 0.046). **f** Similarities in liver FAS expression across experimental groups (main effect injury *p* = 0.528; activity *p* = 0.473; interaction *p* = 0.117). **g** GLUT4 expression unaltered by activity or injury (main effect injury *p* = 0.564; activity *p* = 0.612; interaction *p* = 0.937). **h** FABP4 expression similar across groups (main effect injury *p* = 0.668; activity *p* = 0.723; interaction *p* = 0.715). Full images of all Western Blots is included in Supplementary Fig. 1. Significantly different than †Naïve Restricted. Individual data points represent a single mouse.

Protein expression was evaluated to determine the impact of injury and restricted physical activity on related markers of fatty acid synthesis, oxidation, and glycemic signaling (Fig. 4). The expression of total and phosphorylated acetyl-CoA carboxylase (ACC) within the gastrocnemius muscle was not significantly different across experimental groups (*p* ≥ 0.216; Fig. 4c, d). However, the combination of VML injury and restricted physical activity resulted in a greater ratio of phosphorylated to total ACC, suggesting inactivation of ACC (interaction *p* = 0.046; Fig. 4e). Inactivation of ACC is associated with functional alterations in fatty acid metabolism^29^, therefore, we next evaluated the expression of fatty acid synthase. Fatty acid synthase (FAS) expression in the liver was unaffected by injury or physical activity restriction (*p* ≥ 0.117; Fig. 4f). The expression of GLUT 4 and fatty acid binding protein 4 (FABP4) in the muscle was unaltered (*p* ≥ 0.564, Fig. 4g, h).

### Untargeted metabolomics of the muscle remaining after injury

**Fig. 5:**
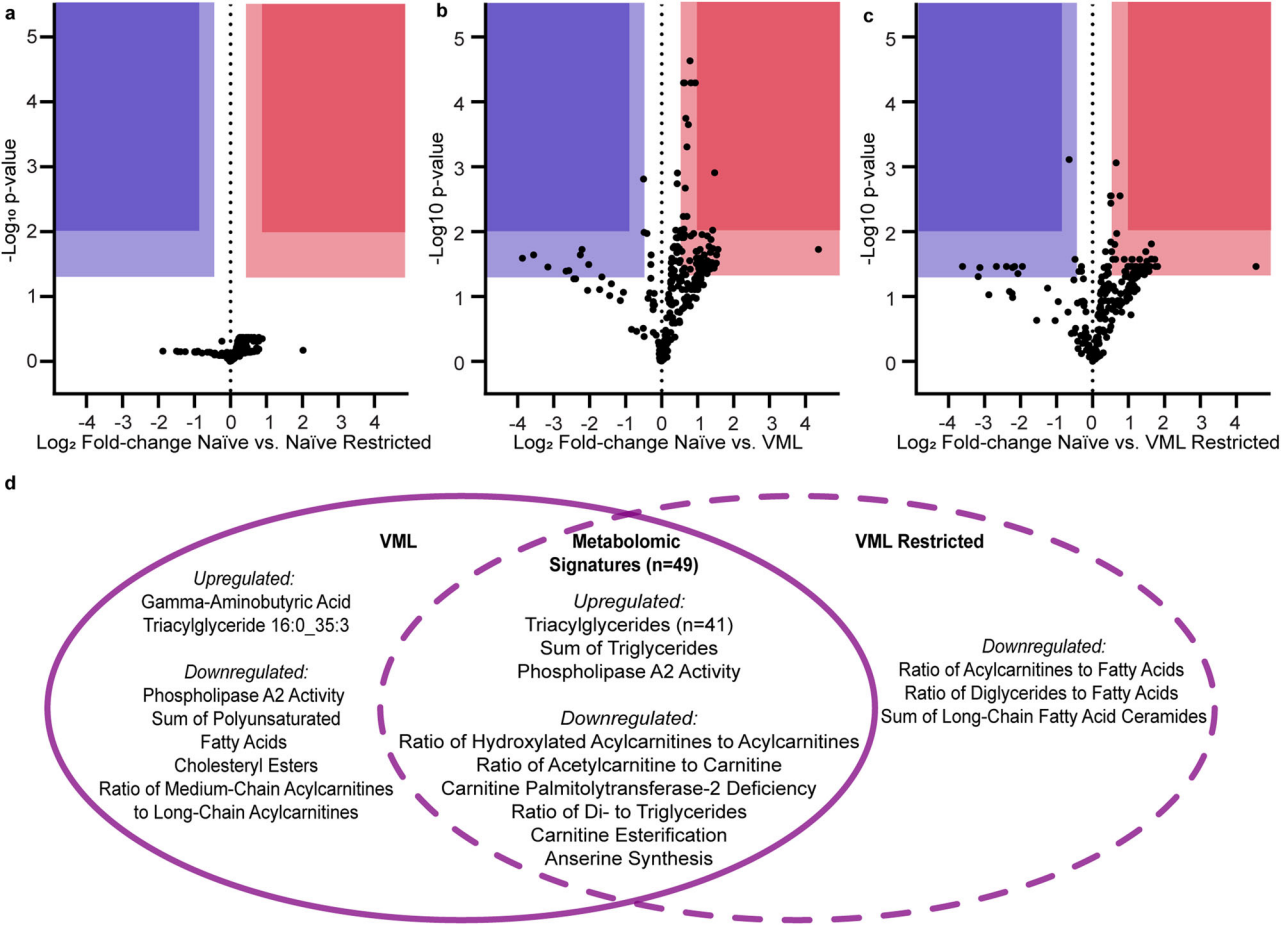
Metabolite expression in the gastrocnemius muscle remaining following VML injury, with and without physical activity restriction, compared to age-matched injury naïve muscle. Evaluation of all experimental groups were compared to the naïve, unrestricted muscle. Volcano plots of **a** restricted activity naïve and injury naïve muscle; **b** VML-injured and injury naïve muscle; and **c** VML-injured restricted activity and injury naïve muscle. Detected metabolites were considered biologically significant and up- or down-regulated at a Log_2_ Fold-Change ≥ 1 or ≤ −1 and statistically significant at a −Log_10_ *p*-value ≥ 1.3 (i.e., *p* ≤ 0.05). Dots represent individual metabolites and metabolite sums and/or ratios (i.e., metabolic signatures). Significantly up- and down-regulated metabolites lie within shaded regions of red and blue, respectively. Darker shaded regions indicate metabolites that are significant at a −Log_10_ *p*-value ≥ 2.0 (i.e., *p* ≤ 0.01). **d** Representative comparison between VML-injured muscle with and without activity restriction. Commonly expressed metabolites posit fatty acid oxidation dysfunction and the resulting accumulation of triglyceride metabolites in the muscle remaining muscle post-VML.

An untargeted metabolomics analysis was performed to evaluate the impact of injury and restricted physical activity on the metabolomic environment of the muscle remaining after VML injury. In this approach, all experimental groups were compared to the naïve muscle. First, 242 metabolic signatures (i.e., metabolites and/or ratios) were detected across the experimental groups. The restriction of physical activity alone did not significantly alter the metabolome, with no metabolic signatures reaching biological or statistical thresholds (Fig. 5a). Significant alterations in the metabolome occurred following VML injury, when compared to injury naïve, with 45 up- and 9 down-regulated metabolic signatures (Fig. 5b). Specifically, 93% of upregulated metabolites following VML were triglycerides containing long-chain fatty acids, suggesting VML injury may promote an adverse accumulation of localized triglycerides. This, in part, may be attributed to a bottlenecking of triglycerides from mitochondrial dysfunction rather than increased fatty acid synthesis. Gamma-aminobutyric acid (GABA) was significantly upregulated 1.5-fold following VML, however, it did not reach significance in any other experimental groups. Results may suggest a heightened mitochondrial GABA release due in part to excess fat accumulation and attempt to increase fatty acid oxidation.

The combination of VML injury and reduced physical activity produced a similar response in the metabolome of the remaining muscle (Fig. 5c). In total, 54 metabolic signatures were significantly up- or down-regulated, with 91% commonly expressed between VML with and without physical activity restriction (Fig. 5d). The downregulated ratios are characteristic of β-oxidation dysregulation, specifically hydroxylated acylcarnitines, carnitine esterification, and the ratio of acetylcarnitine to carnitine. These ratios are indicative of β-oxidation rate and carnitine acyltransferase activity, thus suggesting the impact of VML injury on fatty acid oxidation. Both VML-injured groups exhibited an upregulation in the sum of triglycerides by more than 20-fold, independent of physical activity. The ratio of di- to tri-glycerides was significantly downregulated in both VML-injured groups, further supporting an imbalance of localized triglycerides following VML.

### Muscle enzymatic activity and markers of metabolic syndrome

**Fig. 6:**
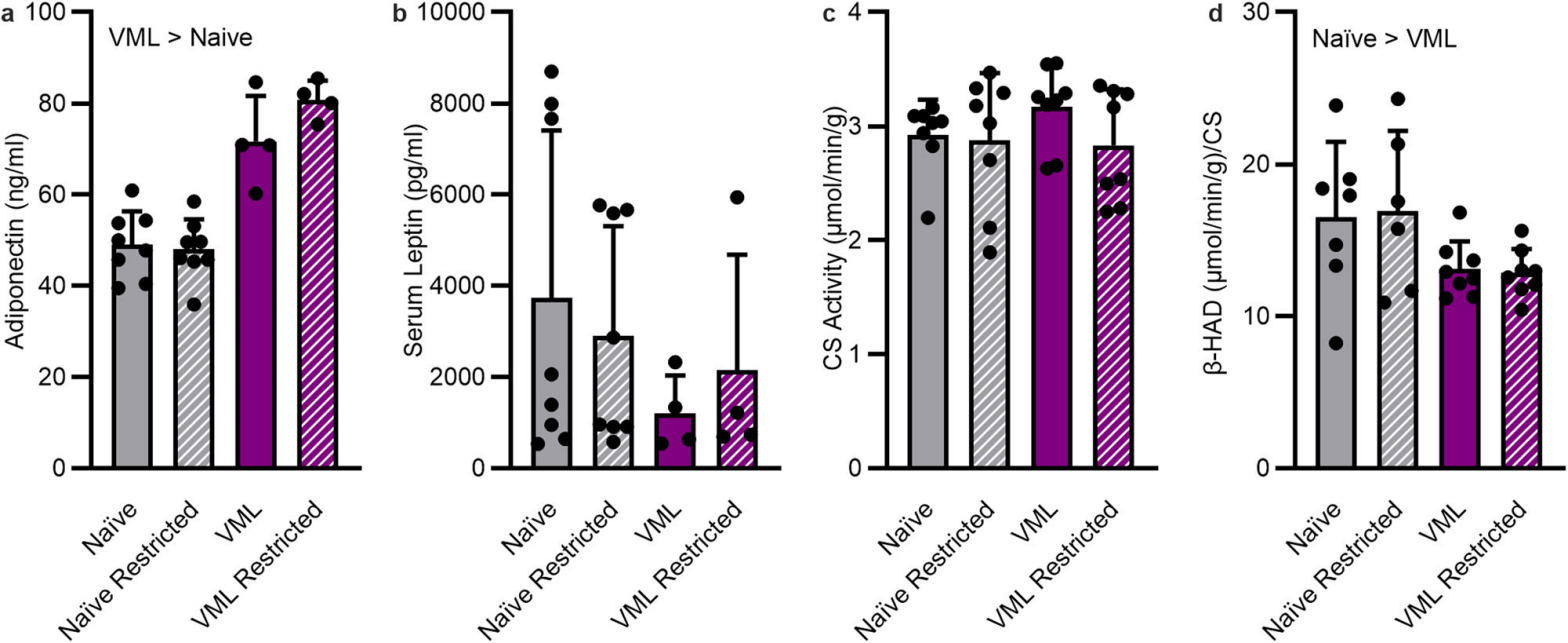
Protein expression of adiponectin in muscle and leptin in serum, and activity of critical mitochondrial enzymes citrate synthase and β-HAD. **a** Higher muscle adiponectin in VML-injured muscles than injury naïve (main effect injury *p* < 0.0001; activity *p* = 0.208; interaction *p* = 0.117). **b** Serum leptin concentrations were unaffected by injury or activity (main effect injury *p* = 0.118; activity *p* = 0.996; interaction *p* = 0.472). **c** Lack of differences in citrate synthase (CS) activity across injury and activity status (main effect injury *p* = 0.524; activity *p* = 0.241; interaction *p* = 0.373). **d** β-HAD activity normalized to CS is blunted by injury (main effect injury *p* = 0.011; activity *p* = 0.948; interaction *p* = 0.808). Individual data points represent a single mouse.

The gastrocnemius muscle and serum were used to evaluate markers of metabolism. Injured mice exhibited ~57% greater levels of adiponectin in the remaining muscle (main effect injury *p* < 0.0001; Fig. 6a). Circulating leptin was not different across groups, however variability was high (*p* ≥ 0.178; Fig. 6b). The activity of citrate synthase (CS) is a surrogate for mitochondrial content and was not affected by injury or physical activity restriction (*p* ≥ 0.241; Fig. 6c). The VML injured muscles exhibited a lower β-HAD activity normalized to CS activity (main effect injury *p* = 0.011; Fig. 6d). β-HAD oxidizes 3-hydroxyaycl-CoA producing a reduced electron equivalent (i.e., NADH) during fatty acid oxidation and a diminished enzyme activity could limit the mitochondria’s ability to efficiently metabolize free-fatty acids.

## Discussion

A thorough understanding of whole-body metabolism and the cellular and metabolomic environment of the remaining uninjured skeletal muscle following both VML injury and physical activity restriction is necessary for efforts to support long-term health in the VML-injured patient population. Clinically, uncoupling physical activity (or inactivity) and recovery from injury is not possible, and currently the full impact of reduced physical activity on VML is only speculated. However, in standardized pre-clinical models of VML there is no change in physical activity levels after injury, quantified through cage ambulation^17,18^ and voluntary running distances^19,30^, when mice are placed in a standard-sized cage, making it difficult to recapitulate the clinical setting. To overcome this discord between the expected clinical population and pre-clinical models, we utilized a restricted physical activity model^16,17,31,32^ in combination with VML injury. We expected that chronic physical activity restriction following VML would worsen the physiologic and metabolic plasticity of the muscle remaining.

As expected, VML injury alone resulted in a ~ 47% loss of muscle torque, ~16% loss of total muscle fiber number, and ~20% loss of muscle mass. Although, surprisingly, there was no impact of restricted activity on these measures of muscle quantity and quality, or changes in the local muscle metabolome in the naïve and VML experimental groups. Specific evaluation of the naïve control, both under standard ambulatory conditions and restricted physical activity, revealed no change in muscle size or torque, which may be a limitation of modeling reductions in physical activity using a smaller cage size. Similar modeling of reduced physical activity has been previously utilized in rodents^16,17,31,32^. The model reduces the size of the livable space. In both the current and past use of the model, there is a 40-75% reduction in daily ambulation, equating to 400–500 m less distance traveled per day and ~90 min of less active time^16,17,31,32^. Across the usages of the model there have been mixed impacts on the muscle, in combination with various pathologies or conditions. However, the preservation of muscle function in the current work was unanticipated. Previously, reductions in maximal torque were apparent after just 6 weeks of restricted physical activity, but these were primarily found in the dorsiflexor muscle group^31^. With a longer period up to 8 weeks of physical activity restriction, there was no impact on muscle torque when activity restriction was implemented immediately following VML^17^; however, this previous work did not have an uninjured group that underwent restricted physical activity for comparison, and evaluations were only on the plantarflexor muscle group. When the tibia was evaluated after physical activity restriction, there was a decrease in the stiffness and ultimate load of the bone^32^, but muscle function was not evaluated. The impact of physical activity restriction on the adjacent tibia suggests that both bone and muscle loading are impaired, the latter of which would be expected with decreased muscle torque. It is possible that the acute and chronic impacts on muscle function and muscle mass are variable following chronically reduced physical activity, as employed herein. Further investigation of physiologic changes in the local metabolism of the muscle needs to be specifically evaluated.

The current work confirms and advances our understanding of the effect of VML injury on whole-body metabolism. Several past studies have reported that whole-body metabolism is altered. Following VML, whole-body metabolic rate is chronically decreased, there is evidence of diurnal metabolic inflexibility, and RER progressively declines^18^. In response to restricted activity following VML, there is a decline in whole-body metabolic rate, while RER increases^17^. Similarly, in the current study, metabolic rate was lower while RER was higher with chronically reduced physical activity following VML injury. The higher RER is reflected in the greater carbohydrate oxidation, similar to the injury naïve group independent of activity. Overall, whole-body metabolic alterations following VML were in large part impacted by physical activity status, similar to observations reported clinically in populations with chronic diseases, such as metabolic syndrome, and following bedrest^28^. It is unclear if alterations in whole-body metabolism with reduced physical activity after VML are regulated by local homeostatic changes in the muscle metabolic environment.

Herein we investigated the muscle metabolic environment in part through evaluation of markers related to fatty acid synthesis and oxidation and metabolomics. Although future work is needed, data suggest that there is decreased fatty acid synthesis when activity is restricted following injury. Synthesized fatty acids are packaged into lipid droplets for storage in various peripheral tissues^33^. Ectopic fatty infiltration following skeletal muscle injury (e.g., rotator cuff injury) is partially facilitated by FABP4, which controls the flux of triglyceride release from lipid droplets for β-oxidation^34^. Alterations in FABP4 expression could hint at excess triglyceride release from lipid droplets. However, FABP4 was unchanged following VML, suggesting VML does not impact lipolytic and lipogenic function. Inside skeletal muscle, ACC plays an important role in both fat synthesis and oxidation. ACC is the rate-limiting enzyme in de novo fatty acid synthesis and is a major regulator of fatty acid oxidation due to its negative feedback on CPT-1 activity (the rate-limiting step of fatty acid oxidation). ACC activity is negatively regulated by AMPK-dependent phosphorylation^35^, a post-translational modification detected herein following VML combined with restricted activity. Reduction in ACC activity can decrease malonyl-CoA concentrations and increase CPT-1 activity that results in decreased fatty acid synthesis and increased fatty acid transport into the mitochondria for oxidation. In theory, this could prompt a greater reliance on fats as a fuel substrate if β-oxidation is completed. However, β-HAD activity was significantly lower in VML-injured animals herein and we’ve previously reported that fat-supported permeabilized muscle fiber mitochondrial respiration is compromised after VML injury^14,21^. Partial or incomplete oxidation of free-fatty acids can lead to an accumulation of tri- and dia-cylglycerols that are associated with cellular stress responses^36–38^. The extent to which incomplete lipid oxidation contributes to the muscle and whole-body pathophysiology after VML injury with or without restricted activity is unclear, but metabolite signature analysis may lend some insight.

To gain further insight into the oxidative dysfunction between VML injury with and without restricted activity we utilized an untargeted metabolomic approach to evaluate metabolite changes. Notably, VML injury alone drove changes in metabolic signatures with over 90% shared outcomes between VML alone and VML combined with restricted activity. Downregulated metabolic signatures are indicative of dysregulation in the carnitine shuttle and subsequent β-oxidation. The ratio between acylcarnitine and carnitine is implicated in mitochondrial myopathy, in part due to excess acylcarnitine accumulation from incomplete fatty acid oxidation^39^. Similarly, carnitine acts as an acute regulator of CPT-1 activity, thus its imbalance would have a downstream impact on β-oxidation. The downregulation of carnitine esterification following VML with and without restricted physical activity implicates CPT-1 as a dysregulated mechanism responsible for the disparity in acylcarnitine and carnitine metabolites. Together with the diminished β-HAD activity in the muscle remaining after VML injury there are possible impairments in fatty acid oxidation flux, and thus suggesting marked accumulation of triglyceride metabolites observed with injury. Dysfunctional lipid oxidation extends beyond the cellular level as whole-body lipid oxidation is diminished with VML injury, and this could induce increases in carbohydrate oxidation due to a change in preferential substrate usage. However, minimal cellular, metabolite, or enzymatic (i.e., GLUT4, CS activity) outcomes in support of this notion are observed following VML injury. Increased whole-body glucose tolerance following VML injury presents an interesting finding to support a synergistic relationship between whole-body fat and carbohydrate oxidation, in that alterations in fatty acid oxidation in the remaining muscle following VML may be driving changes in whole-body carbohydrate metabolism.

Some attributes of whole-body metabolism that change after VML injury are mirrored by the metabolism in the VML-injured skeletal muscle, although a causal link remains unclear. Oxygen consumption rate in the remaining muscle following VML is disproportionately reduced in relation to magnitude of lost muscle^19^. As a result of mitochondrial dysfunction, fat-supported respiratory flux is impaired up to two weeks after VML with concomitant decreases in electron conductance^14^. β-oxidation occurs inside the mitochondria and therefore a muscle’s ability to utilize fats as a fuel source could be influenced by mitochondrial content. CS activity, responsible for controlling the flux of metabolites into the citric acid cycle, is a strong surrogate of mitochondrial content^40^. Mitochondrial content is increased up to three weeks following VML compared to more acute timepoints, suggesting there is a physiologic delay after injury impacting the metabolic dysfunction^41^, yet chronically, there were not differences in CS activity detected here with VML injury with or without activity restriction. Histological evaluation of NADH-positive fibers is another approach to gauge the general oxidative capacity of a skeletal muscle that also allows for muscle region-specific differentiation. Results from this study indicate a more pronounced effect of VML injury on the oxidative capacity of muscle at the defect and border regions as compared to the remaining muscle region. This agrees with a previous study that analyzed mitochondrial network organization at different distances from the VML-defect and reported an increasing disruption to the mitochondrial network moving from the distant remaining muscle to the border region to the defect region^19^. Mitochondrial enzyme activity and permeabilized muscle fiber respiration analyses may under-represent the metabolic detriments following VML injury because they tend to rely upon fibers collected from the remaining muscle. The totality of the effect of VML injury on the collective muscle, and potentially the whole body, remains an open question.

Several circulating and local muscle factors were evaluated to identify links between the muscle and whole-body metabolism changes. Adiponectin is a known activator of AMPK and aids in the regulation of multiple metabolic processes, including glycemic control and fatty acid oxidation^42^. Skeletal muscle expression of adiponectin is increased in times of high oxidative stress as a protective mechanism to counteract excessive oxidative damage^43^. Similarly, hyperleptinemia has also been reported in times of high oxidative stress in metabolic diseases, such as obesity and type II diabetes^44^. Elevated adiponectin within the muscle remaining after VML could contribute to greater whole-body glucose tolerance. However, there was a lack of parallel increases in circulating leptin and muscle GLUT4 expression, suggesting whole-body glucose tolerance was not influenced by an increase in transporter-mediated uptake or quantity. Notably, clinical pathologies associated with impaired muscle function (e.g., aging) are characterized by high serum adiponectin levels that, can have paradoxical detrimental effects on muscle regeneration^45^. It is unclear if heightened adiponectin, and subsequent improvements in glucose tolerance, in the remaining muscle following VML is a favorable or unfavorable secondary outcome to the injury.

This work evaluated the whole-body, cellular, and metabolomic environment and plasticity of the remaining uninjured skeletal muscle following both VML injury and reduced physical activity. Clinically, there is an expected loss of physical activity (i.e., sedentarism) immediately following VML injury, which could impact the long-term development of various comorbidities^46^, such as type II diabetes, metabolic syndrome, obesity, etc. Remarkably, there was a broad lack of changes, particularly in the remaining muscle metabolism, due to physical activity restriction, with most changes being linked to VML injury alone. This work adds to the understanding of the natural sequela of VML injury, indicating VML limits the ability of the muscle remaining to oxidize fatty acids resulting in a possible accumulation of triglycerides; however, further investigation is warranted. Future work needs to continue to evaluate the impact of VML-induced alterations in fatty acid metabolism and how fat accumulation acutely and chronically after injury may affect long term function and plasticity of the muscle remaining.

## Methods

### Ethical approval and experimental design

All protocols were approved by the Institutional Animal Care and Use Committee at the University of Minnesota (#2110-39493A), in compliance with the Animal Welfare Act, the Implementing Animal Welfare Regulations and in accordance with the principles of the Guide for the Care and Use of Laboratory Animals. Adult male C57Bl/6 J mice (*n* = 40; 8–12 per group) were purchased from Jackson Laboratories (Stock #000664; Bar Harbor, ME). Mice were given at least a one-week acclimation period prior to study initiation. Mice were housed on a 12-h light-dark cycle (light phase begins at 06:00) with *ad libitum* access to chow (LabDiet #5053, Land O’ Lakes, Inc.) and water.

At ~13 weeks of age, mice were randomized to VML injury or served as age-matched injury naïve controls. Mice were further randomized to standard or restricted cages^16,17^. As we have previously used, restricted cages (12.5 × 8.5 × 6.3 cm) reduce physical activity by ~50% and alter whole-body metabolism within the first week following restriction, compared to standard cages (28 x 18 x 12.5 cm)^16,17^. At 6 weeks following VML, whole-body metabolic and physical activity measurements were evaluated for all mice using the Comprehensive Lab Animal Monitoring System (CLAMS; Columbus Instruments). Glucose tolerance was evaluated at 7 weeks following VML or study start. Terminally 8 weeks post-VML ( ~ 21 weeks of age) in vivo muscle function was assessed, and skeletal muscles, liver, and serum were subsequently extracted. Gastrocnemius muscles were weighed, cut into thirds (proximal, mid, distal sections), frozen in liquid nitrogen, and stored at −80^o^C for later analyses. A subset of the mid-portion of the gastrocnemius muscle, encompassing the VML defect, was saved in OCT, frozen in isopentane cooled with liquid nitrogen, and stored at −80^o^C for histological evaluation. A portion of liver tissue was weighed, frozen in liquid nitrogen, and stored at −80^o^C. Blood was clotted and centrifuged at 2000 rpm for 15 min at 4°C, serum was collected and stored at −20^o^C. Mice were euthanized with pentobarbital ( > 100 mg/kg; s.q.).

### Volumetric muscle loss (VML) surgical procedure

As described previously^19,47^, a full thickness VML injury was surgically created to the muscles of the posterior hindlimb compartment (gastrocnemius, soleus, plantaris muscles). Mice received buprenorphine SR (1.2 mg/kg; s.q.) approximately 2 h prior to surgery for pain management. Mice were anesthetized by isoflurane inhalation ( ~ 2.0%) under aseptic surgical conditions. Briefly, a posterior-lateral incision was created through the skin to reveal the gastrocnemius muscle. Blunt dissection isolated the posterior muscle compartment, and a metal plate was inserted between the tibia and the deep aspect of the soleus. A 4-mm punch biopsy (19.1 ± 1.6 mg, ~15% volume loss of muscle) was performed on the middle third of the muscle compartment. Any bleeding was stopped with light pressure. Skin incisions were closed with 6–0 PGA suture and animals were monitored through recovery.

### Evaluation of physical activity and whole-body metabolism

Physical activity and whole-body metabolic assessments were conducted as previously described^17,18^ using the CLAMS system (Columbus Instruments) and data examination tool (Clax, v2.2.15; Columbus Instruments, Columbus, OH, USA). Physical activity data were collected over 10-sec increments and metabolic data were collected in 10-min intervals over 24 h and processed. MATLAB (version R2020a, MathWorks, Natick, MA, USA) was used to calculate metabolic rate, RER, and carbohydrate and lipid oxidation rate averages over 24-h and 12-h active and inactive periods, and to evaluate 24-h moving averages for RER. Area under the curve (AUC) for RER was calculated over 24-h and 12-h active and inactive periods. Delta RER (Δ RER) was determined as the difference between average RER across the 12-h active and 12-h inactive periods. Mice were given a 24-h acclimation period prior to the data collection period.

### Glucose tolerance test

As previously described^17^, at 7 weeks following VML (one week prior to harvest), glucose tolerance testing was performed after a 6-h fast. Baseline blood glucose was obtained from the lateral tail vein, nicked with a 20-G needle, with a glucometer (Freestyle Lite, Abbott). Following injection of D-glucose saline solution (Sigma #G7021; 2 mg/g, i.p.), glucose measurements were obtained at 15−, 30−, 45−, 60−, 90−, and 120-min following injection. The glucometer measured a range of glucose values up to 500 mg/dl; readings above this range were recorded, plotted, and analyzed as 500 mg/dl. Mice were continuously monitored, and any additional bleeding was stopped with light pressure. Following testing, mice were returned to home cages with *ad libitum* access to food and water.

### In vivo muscle function

Muscle function of the posterior compartment was evaluated 8 weeks following VML or study start (naïve) as previously described^19,47^. Briefly, mice were anesthetized using inhaled isoflurane (1.5–2.0%) and body temperature was maintained at 37^o^C. Mice were subsequently positioned on the right side with the left foot attached to the footplate of the dual‐mode muscle lever system (300C-LR; Aurora Scientific, Aurora, Ontario, Canada). The knee and hip were stabilized at 90^o^. First, the common peroneal nerve was severed to isolate stimulation to the posterior compartment. Maximal isometric torque was evaluated by stimulating the sciatic nerve using Platinum-Iridium percutaneous needle electrodes. Torque frequency was conducted (5, 10, 20, 40, 60, 80, 100, 150, and 200 Hz) and expressed as mN·m per kg body weight.

### Histological analyses

Histological evaluation was performed in the mid-belly of the gastrocnemius muscle. Ten-µm serial cross-sections were obtained using a Leica cryostat and microtome, and subsequently stained for hematoxylin and eosin to evaluate myofiber number and morphology and NADH-tetrazolium reductase to assess percentage of oxidative myofibers.

The NADH-TR staining was performed as previously described by incubating tissues at 37°C for 20 min in a solution containing 0.2 M Tris, 1.5 mM NADH, and 1.5 mM nitro blue tetrazolium, as previously described^18^. Sections were washed, dehydrated, and cleared in xylenes. Brightfield images were acquired using the TissueScope LE slide scanner (Huron Digital Pathology, St. Jacobs, ON, Canada) using a 20X objective (0.75 NA, 0.5 µm/pixel resolution). Following imaging, HuronViewer (Huron Digital Pathology) was used to export three standardized non-overlapping regions of interest (ROI) for each muscle. All ROIs were standardized to 1000 × 1000 µm. Regions encompassed the VML defect, the border of the defect which included the defect and remaining muscle, and the remaining muscle tissue. The corresponding three ROI areas were also obtained in muscles of the injury naïve groups. All ROIs were systematically evaluated in NADH-stained muscles. Data from these ROIs were averaged across the gastrocnemius muscle and compared across groups.

Analyses of muscle sections were conducted using Fiji^48^. The multipoint tool was used to manually count myofiber numbers and types, in addition to the number of darkly and lightly stained muscle fibers for NADH-stained muscle. Darkly stained fibers were classified as positive for NADH, representing high metabolic activity, while lightly stained fibers were classified as negative for NADH, representing low metabolic activity. Investigators were blinded during all imaging and post-imaging analyses.

### Biochemical analyses

The proximal gastrocnemius muscle, liver, and serum were used in biochemical analyses. Gastrocnemius muscles and liver were homogenized in 10 mM phosphate buffer at a ratio of 1:10 (mg/µl) with the addition of 1:100 protease and phosphatase inhibitors. Total protein content was analyzed using the Protein A280 setting on a NanoDrop One spectrophotometer (Thermo Scientific) in triplicate and averaged.

Muscle adiponectin concentrations were assessed in duplicate (Milliplex #MADPNMAG-70K-01); plates were read using a Bio-Plex 200 system (Bio-Rad Laboratories; Hercules, CA, USA). Prepared standards were validated within an acceptable recovery range of 70–130% (observed concentration/expected concentration). Serum leptin concentrations were evaluated by ELISA (R&D Systems; #MOB00B), according to the manufacturer’s protocol. Values were excluded if the observed concentration was below the minimum detectable concentration of 2.58 pg/ml.

Immunoblot analyses of muscle and liver were performed by separating 20–50 µg of protein by 4–15% Criterion TGX Stain-Free Gel (Bio-Rad), transferring protein onto a PVDF membrane, and immunoblotting. Primary antibodies were used to probe for acetyl-CoA carboxylase (Cell Signaling #3676; Lot #12; RRID: AB2219397; 1:000), phospho-acetyl-CoA carboxylase (Cell Signaling #3661; Lot #10; RRID:AB330337; 1:1000), fatty acid synthase (Cell Signaling #C20G5, Lot #7; RRID:AB_2100796; 1:1000), FABP4 (Abcam #ab92501; Lot#1006255-21; RRID:AB_10562486; 1:1000) and GLUT4 (Millipore Sigma #07-1404; Lot #3829399; RRID:AB1587080; 1:2000). Antibodies were detected using a corresponding host- and isotype-specific horseradish peroxidase conjugated secondary antibody (Cell Signaling #7074, 1:1000). Immunoblots were blocked with 5% nonfat milk, and primary and secondary antibodies were diluted in 5% nonfat milk, followed by incubation in Clarity Max ECL Western Blotting Substrate for protein detection (Bio-Rad). Immunoblots were visualized with stain-free and chemiluminescent imaging on a ChemiDoc System (Bio-Rad) for total lane protein and band intensity quantification, respectively^49^. The intensity of each band was normalized to total protein in each respective lane using Bio-Rad Laboratories Image Lab software (Hercules, CA). The gastrocnemius muscle of injury naïve mice was used for relative comparison across experimental groups on all immunoblots, full images of all Western Blots is included in Supplementary Fig. 1.

The muscle was used to evaluate mitochondrial content^15,19^ and β-hydroxyacyl-CoA dehydrogenase (β-HAD)^21,50^, as previously described. Briefly, the muscle homogenate was further diluted with10 mM phosphate buffer to a final ratio of 1:40 (mg/µl). Mitochondrial content was determined by citrate synthase (CS) activity by evaluating the reduction of 5,5’-dithio-bis(2-nitrobenzoic) acid (DTNB) over time, at 412 nm evaluated using a spectrophotometer. The activity of β-HAD was determined by incubating muscle homogenate in a buffer containing 100 mM triethanolamine, 451 µM β-nicotinamide adenine dinucleotide and 5 mM ethylenediaminetetraacetic acid (EDTA). The enzyme activity of β-HAD was normalized to mitochondrial content (i.e., CS enzyme activity).

### Measurement of tissue metabolites

Metabolomics measurements were obtained using the MxP Quant 500 kit (Biocrates Life Sciences AG, Innsbruck, Austria) following the manufacturer’s protocol for tissue samples^17^. Distal gastrocnemius muscles ( ~ 50 mg) were placed in 2 mL Precellys CKMix lysis tubes (Bertin Corp., Rockville, MD, USA) and diluted threefold with cold isopropanol. Samples were homogenized using a Precellys bead beater set to 4°C three times for 30 s at 5800 rpm, 30 s pause between pulses. Samples were centrifuged for five min at 10,000 x *g* and the supernatant was transferred to a new vial. Samples were analyzed in two batches using a 56-well and 96-well based sample preparation plate, respectively. The 56-well plate was analyzed using a Sciex QTRAP 5500 mass spectrometer (Sciex, Framingham, MA, USA) coupled to a Shimadzu LC-20AD XR (Shimadzu USA Manufacturing Inc., Canby, OR, USA) liquid chromatography platform. The 96-well plate was analyzed using an Agilent 6495 C triple quadrupole platform coupled with an Agilent Infinity II liquid chromatography system (Agilent, Santa Clara, CA, USA). Small molecule metabolites were measured by liquid chromatography-tandem mass spectrometry (LC-MS/MS). Lipids and hexoses were measured by flow injection analysis-tandem mass spectrometry (FIA-MS/MS). The metabolomics measurement technique is described in detail by patents EP1875401B1^51^ and EP1897014B1^52^. Data was processed using the WebIDQ online software (Biocrates Life Sciences AG, Innsbruck, Austria). Metabolite concentrations were adjusted using a tissue correction factor and between-plate concentrations were normalized to an internal quality control sample.

### Statistical analyses

All statistical analyses were conducted using JMP (version 16.0.0, SAS Institute, Inc.) and R programming language^53^. Two-way ANOVA with Tukey’s honest significant (HSD) difference *post hoc* test was used to evaluate differences across injury and physical activity status groups for whole-body metabolism (24- and 12-h metabolic rate, RER, lipid oxidation, carbohydrate oxidation) and ambulatory distance, in vivo twitch and maximal isometric torque and corresponding contractile curve analyses (e.g., average rates of contraction and relaxation, half relaxation time, time to peak), terminal body and muscle masses, protein expression, enzyme activity and signaling markers in muscle, serum, and/or liver. The RER AUC was compared by one-way ANOVA with Tukey’s HSD *post hoc* test. Three-way ANOVA with Tukey’s HSD *post hoc* test evaluated differences across injury status, physical activity status, and time during the glucose tolerance test. All data is shown as mean ± SD unless otherwise noted. Significance was set a priori at *p* < 0.05 all exact statistical outcomes are presented in Supplementary Table 1.

For metabolomics analyses, metabolite measurements with greater than 20% missing values among the sample cohort were excluded from the analysis. Missing values were imputed using a K-Nearest Neighbors (*k* = 10) approach using the *impute* R package^54^. Metabolomic concentrations were Log transformed to improve the normality assumption for statistical modeling. Statistical differences between injury-movement systems were analyzed using a generalized linear model approach controlling for batch-wise variation. Model effect estimates and statistical *p*-values were estimated for each metabolite independently. A Benjamini-Hochberg *p*-value adjustment was applied to control for multiple comparisons^55^.

## Supplementary information

SupplementaryInformation

## Data Availability

No datasets were generated or analysed during the current study.
