# Supplementary material for: Limitations in metabolic plasticity after traumatic injury are only moderately exacerbated by physical activity restriction
Source: NPJ Metab Health Dis. 2024 Apr 6;2:4. doi: 10.1038/s44324-024-00006-5 (PMC11486518; doi:10.1038/s44324-024-00006-5)
Supplement: Supplementary file 1 — SupplementaryInformation [file 44324_2024_6_MOESM1_ESM.pdf]

Limitations in metabolic plasticity after traumatic injury are only moderately exacerbated by physical activity restriction

Supplementary Information

Angela S. Bruzina,[1⁋] Christiana J. Raymond-Pope,[1⁋] Kevin J. Murray,[2] Thomas J. Lillquist,[1] Katelyn M. Castelli,[3] Shefali R. Bijwadia,[1] Jarrod A. Call[3,4] & Sarah M. Greising[1]*

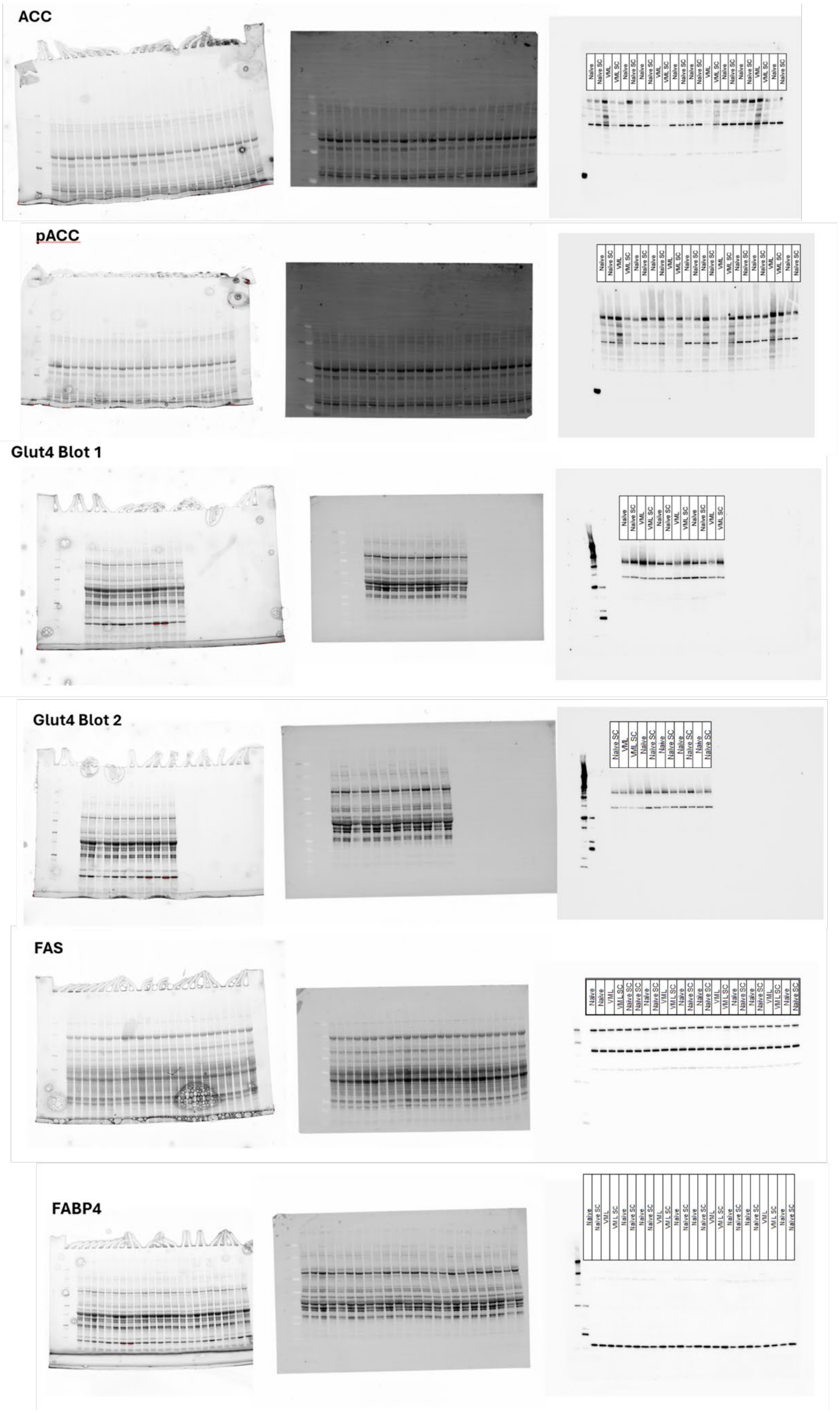

Supplementary Figure 1. Western blot images of stain-free gels, stain-free blots, and chemiluminescence blots, respectively. ACC (acetyl-coa carboxylase), pACC (phosphorylated acetyl-coa carboxylase), GLUT4

(glucose transporter type 4), FAS (fatty acid synthase), FABP4 (fatty acid-binding protein 4). Each chemiluminescence blot is labeled with each individual row corresponding with experimental group. Naïve SC (small cage, naïve with restricted activity), VML SC (small cage, volumetric muscle loss with restricted activity).

Supplementary Table 1: Statistical Summary Table

Experimental question number; multiple tests of the same question are shown in different rows	Finding/ conclusion	Experimental variable	Timepoint	Mean value	Standard Deviation (SD)	n	Exact P value	Figure/table in which data are presented	Units	Data comparisons	Statistical test	Comments
**1. Measures of body mass and injured gastrocnemius muscle characteristics**	No differences across groups	Naïve Unrestricted	Pre-VML	29.0	1.8	8	0.494	Table 1	g	All groups compared	Two-way ANOVA	Main effect of injury p=0.101; activity p=0.433
A. Pre-VML Body Mass		VML Unrestricted		27.4	2.3	12						Interaction injury x activity p=0.494
		Naïve Restricted		28.0	1.3	8						
		VML Restricted		27.3	2.3	12						
B. Terminal Body Mass	All mice with restricted activity (naïve and VML) had lower body mass than mice with unrestricted activity, independent of injury status.	Naïve Unrestricted	8 weeks post-VML	30.9	1.8	8	**Main effect of activity p=0.026**	Table 1	g	All groups compared	Two-way ANOVA	Main effect of injury p=0.219; **activity p=0.026**
		VML Unrestricted		30.5	2.7	12						Interaction injury x activity p=0.094
		Naïve Restricted		27.8	1.6	8						
		VML Restricted		30.1	2.7	12						
C. Change in Body Mass (%)	Percent change in body mass was greater in all VML-injured mice compared to no injury.	Naïve Unrestricted		6.7	3.1	8	**Main effect of injury p<0.0001**	Table 1	%	All groups compared	Two-way ANOVA	**Maine effect of injury p<0.0001**; activity p=0.066
		VML Unrestricted		11.6	5.3	12						Interaction injury x activity p=0.123
		Naïve Restricted		-0.7	1.9	8						
		VML Restricted		11.1	7.8	12						
D. Left Gastrocnemius Mass	VML-injured mice had lower gastrocnemius masses than naïve mice, independent of activity level.	Naïve Unrestricted		181.6	10.5	8	**Main effect of injury p<0.0001**	Table 1	mg	All groups compared	Two-way ANOVA	**Main effect of injury p<0.0001**; activity p=0.116
		VML Unrestricted		139.7	22.9	12						Interaction injury x activity p=0.171
		Naïve Restricted		166.4	10.2	8						
		VML Restricted		138.6	12.1	12						
E. Left Gastrocnemius Mass Normalized to Body Mass	VML-injured mice had lower gastrocnemius masses normalized to body mass than naïve mice, independent of activity level.	Naïve Unrestricted		5.89	0.35	8	**Main effect of injury p<0.0001**	Table 1	mg/g	All groups compared	Two-way ANOVA	**Main effect of injury p<0.0001**; activity p=0.218
		VML Unrestricted		4.58	0.62	12						Interaction injury x activity p=0.344
		Naïve Restricted		6.18	0.24	8						
		VML Restricted		4.62	0.20	12						
**2. Measures of posterior hindlimb compartment contractile function**	VML injury resulted in lower twitch torque, with the restriction of activity partially rescuing the deficit in twitch torque.	Naïve Unrestricted	8 weeks post-VML	134.7	18.0	8	**Main effect injury p<0.0001; main effect activity p=0.034**	Fig. 1A	mN*m/kg	All groups compared	Two-way ANOVA	**Main effect of injury p<0.0001**; main effect of activity p=0.034
A. Twitch Torque		VML Unrestricted		54.3	24.2	12						Interaction injury x activity p=0.895
		Naïve Restricted		151.1	24.8	8						
		VML Restricted		68.9	18.9	12						
B. Maximal Isometric Torque	VML-injured mice had lower maximal isometric torque compared to naïve.	Naïve Unrestricted		538.8	67.4	8	**Main effect injury p<0.0001**	Fig. 1B	mN*m/kg	All groups compared	Two-way ANOVA	**Main effect of injury p<0.0001**; main effect of activity p=0.518
		VML Unrestricted		297.3	90.5	12						Interaction injury x activity p=0.958
		Naïve Restricted		581.8	100.9	8						
		VML Restricted		315.2	70.0	12						
C. Time to Peak Twitch	VML injury resulted in a lower time to peak twitch.	Naïve Unrestricted		0.028	0.002	8	**Main effect injury p=0.0003**	Table 2	s	All groups compared	Two-way ANOVA	**Main effect of injury p=0.0003**; main effect of activity p=0.872
		VML Unrestricted		0.023	0.003	12						Interaction injury x activity p=0.703
		Naïve Restricted		0.027	0.004	8						
		VML Restricted		0.023	0.005	12						
D. Twitch : Tetanus Ratio	VML injury resulted in a lower twitch to tenatus ratio	Naïve Unrestricted		0.26	0.05	8	**Main effect injury p=0.0002**	Table 2	A.U.	All groups compared	Two-way ANOVA	**Main effect of injury p=0.0002**; main effect of activity p=0.111
		VML Unrestricted		0.18	0.05	12						Interaction injury x activity p=0.105
		Naïve Restricted		0.26	0.04	8						
		VML Restricted		0.23	0.03	12						
E. 1/2 Relaxation Time	VML injury resulted in a lower twitch half-relaxation time	Naïve Unrestricted		0.020	0.001	8	**Main effect injury p<0.0001**	Table 2	s	All groups compared	Two-way ANOVA	**Main effect of injury p<0.0001**; main effect of activity p=0.663
		VML Unrestricted		0.015	0.003	12						Interaction injury x activity p=0.561
		Naïve Restricted		0.019	0.004	8						
		VML Restricted		0.015	0.003	12						
F. +dP/dt	VML injury resuted in a longer rate of force development	Naïve Unrestricted		235.3	31.2	8	**Main effect injury p=0.023**	Table 2	mN*m/s	All groups compared	Two-way ANOVA	**Main effect of injury p=0.023**; main effect of activity p=0.977
		VML Unrestricted		189.1	70.9	12						Interaction injury x activity p=0.838
		Naïve Restricted		239.1	58.7	8						
		VML Restricted		184.1	64.2	12						
G. -dP/dt	VML injury resuted in a longer rate of relaxation	Naïve Unrestricted		-340.6	62.4	8	**Main effect injury p<0.0001**	Table 2	mN*m/s	All groups compared	Two-way ANOVA	**Main effect of injury p<0.0001**; main effect of activity p=0.851
		VML Unrestricted		-194.7	78.3	12						Interaction injury x activity p=0.645
		Naïve Restricted		-333.5	58.2	8						
		VML Restricted		-211.6	86.4	12						

**3. Measures of physical activity and whole-body metabolism with or without physical activity restriction**	Restriction in physical activity decreased daily ambulation	Naïve Unrestricted	6 weeks post-VML	1321	281	8	**Main effect of activity p<0.0001**	Fig. 2A	m/day	All groups compared	Two-way ANOVA	Main effect of injury p=0:2406; **activity p<0.0001**
A. Ambulation (m/day)		VML Unrestricted		773	123	12						Interaction injury x activity p=0.924
		Naïve Restricted		1222	371	8						
		VML Restricted		863	165	12						
B. 24-h Metabolic Rate	VML-injured mice with restricted activity have lower 24-hr metabolic rate compared to VML alone.	Naïve Unrestricted	6 weeks post-VML	16.8	0.6	8	**Interaction injury x activity p=0.033**	Fig. 2B	kcal/hr	All groups compared	Two-way ANOVA	Main effect of injury p=0.5662; activity p=0.017
		VML Unrestricted		17.6	1.0	12						**Interaction injury x activity p=0.033**
		Naïve Restricted		16.7	1.1	8						
		VML Restricted		16.3	1.0	12						
C. 12-h Inactive Metabolic Rate	VML-injured mice with restricted activity have lower metabolic rate in the 12-hr inactive period compared to VML alone.	Naïve Unrestricted	6 weeks post-VML	14.9	0.6	8	**Interaction injury x activity p=0.037**	Fig. 2B	kcal/hr	All groups compared	Two-way ANOVA	Main effect of injury p=0.559; activity p=0.059
		VML Unrestricted		15.0	0.9	12						**Interaction injury x activity p=0.037**
		Naïve Restricted		15.1	0.9	8						
		VML Restricted		14.0	0.5	12						
D. 12-h Active Metabolic Rate	The restriction of activity results in a lower metabolic rate in the 12-hr active period, regardless of injury.	Naïve Unrestricted	6 weeks post-VML	18.4	0.9	8	**Main effect of activity p=0.029**	Fig. 2B	kcal/hr	All groups compared	Two-way ANOVA	Main effect of injury p=0.278; **activity p=0.029**
		VML Unrestricted		19.8	1.4	12						Interaction injury x activity p=0.084
		Naïve Restricted		18.1	1.5	8						
		VML Restricted		17.8	1.1	12						
E. 24-h RER	The restriction of activity results in a higher 24-hr RER.	Naïve Unrestricted	6 weeks post-VML	0.83	0.02	8	**Main effect of activity p=0.001**	Fig. 2C	Ratio	All groups compared	Two-way ANOVA	Main effect of injury p=0.187; **activity p=0.001**
		VML Unrestricted		0.88	0.02	12						Interaction injury x activity p=0.0.88
		Naïve Restricted		0.86	0.01	8						
		VML Restricted		0.88	0.03	12						
F. 12-h Inactive RER	The restriction of activity results in a higher RER in the 12-hr inactive period.	Naïve Unrestricted	6 weeks post-VML	0.80	0.02	8	**Main effect of activity p=0.001**	Fig. 2C	Ratio	All groups compared	Two-way ANOVA	Main effect of injury p=0.559; **activity p=0.001**
		VML Unrestricted		0.85	0.02	12						Interaction injury x activity p=0.419
		Naïve Restricted		0.81	0.02	8						
		VML Restricted		0.86	0.04	12						
G. 12-h Active RER	The restriction of activity, regardless of injury, resulted in a higher RER in the 12-hr active period compared to naïve.	Naïve Unrestricted	6 weeks post-VML	0.86	0.02	8	**Interaction injury x activity p=0.047**	Fig. 2C	Ratio	All groups compared	Two-way ANOVA	**Interaction injury x activity p=0.047**
		VML Unrestricted		0.90	0.03	12						
		Naïve Restricted		0.89	0.02	8						
		VML Restricted		0.93	0.03	12						
H. 24-hr RER AUC	VML resulted in greater 24-hr RER AUC; The restriciton of activity resulted in greater 24-hr RER AUC	Naïve Unrestricted	6 weeks post-VML	18.83	1.09	8	**Main effect of activity p<0.0001; injury p<0.0001**	Fig. 2E	A.U.	All groups compared	One-way ANOVA	**Main effect of injury p<0.0001; activity p<0.0001**
		VML Unrestricted		21.15	1.26	12						
		Naïve Restricted		22.21	1.56	8						
		VML Restricted		27.13	1.99	12						
I. 12-hr Inactive RER AUC	The restirciton of activity resulted in a greater RER AUC in the 12-hr inactive period	Naïve Unrestricted	6 weeks post-VML	8.11	0.75	8	**Main effect of activity p<0.0001**	Fig. 2E	A.U.	All groups compared	One-Way ANOVA	Main effect of injury p=0.426; **activity p<0.0001**
		VML Unrestricted		7.73	0.84	12						
		Naïve Restricted		10.19	1.15	8						
		VML Restricted		12.12	1.33	12						
J. 12-hr Active RER AUC	VML injury resulted in greater RER AUC in the 12-hr active period. The restriciton of activity resulted in greater RER AUC in the 12-hr active	Naïve Unrestricted	6 weeks post-VML	10.71	0.79	8	**Main effect of activity p<0.0001; injury p<0.0001**	Fig. 2E	A.U.	All groups compared	One-way ANOVA	**Main effect of injury p<0.0001; activity p<0.0001**
		VML Unrestricted		13.42	0.94	12						
		Naïve Restricted		12.02	1.06	8						
		VML Restricted		15.00	1.49	12						
K. Δ RER	VMl injury resulted in a greater Δ RER from the inactive to active period	Naïve Unrestricted	6 weeks post-VML	0.036	0.04	8	**Main effect of injury p=0.030**	Fig. 2F	Ratio	All groups compared	Two-way ANOVA	**Main effect of injury p=0.030**; activity p=0.058
		VML Unrestricted		0.078	0.19	12						Interaction injury x activity p=0.269
		Naïve Restricted		0.025	0.07	8						
		VML Restricted		0.039	0.03	12						
L. 24-h Lipid Oxidation	No differenes in whole-body lipid oxidation in the 24-hr period across experimental groups	Naïve Unrestricted	6 weeks post-VML	1.31	0.48	8	0.365	Fig. 2G	g/min	All groups compared	Two-way ANOVA	Main effect of injury p=0.068; activity p=0.894
		VML Unrestricted		1.41	0.34	12						Interaction injury x activity p=0.365
		Naïve Restricted		1.19	0.36	8						
		VML Restricted		1.06	0.39	12						
M. 12-h Inactive Lipid Oxidation	No differences in whole-body lipid oxidation in the 12-hr inactive period across experimental groups	Naïve Unrestricted	6 weeks post-VML	1.51	0.52	8	0.868	Fig. 2G	g/min	All groups compared	Two-way ANOVA	Main effect of injury p=0.334; activity p=0.857
		VML Unrestricted		1.56	0.34	12						Interaction injury x activity p=0.868
		Naïve Restricted		1.41	0.41	8						
		VML Restricted		1.41	0.34	12						
N. 12-h Active Lipid Oxidation	VML injury resulted in lower whole-body lipid oxidation in the 12-hr active period	Naïve Unrestricted	6 weeks post-VML	1.14	0.49	8	**Main effect of injury p=0.020**	Fig. 2G	g/min	All groups compared	Two-way ANOVA	**Main effect of injury p=0.020;** activity p=0.707
		VML Unrestricted		1.29	0.40	12						Interaction injury x activity p=0.157
		Naïve Restricted		1.01	0.34	8						
		VML Restricted		0.76	0.46	12						
O. 24-h Carbohydrate Oxidation	Restriction of activity following VML resulted in greatest whole-body carbodhyrate oxidation across 24-hr period	Naïve Unrestricted	6 weeks post-VML	4.77	0.71	8	**Interaction injury x activity p=0.001**	Fig. 2H	g/min	All groups compared	Two-way ANOVA	**Main effect of injury p=0.004;** activity p=0.083
		VML Unrestricted		4.43	0.71	12						**Interaction injury x activity p=0.001**
		Naïve Restricted		4.70	0.70	8						
		VML Restricted		5.89	0.82	12						
P. 12-h Inactive Carbohydrate Oxidation	No differences in whole-body carbohydrate oxidation in the 12-hr inactive period	Naïve Unrestricted	6 weeks post-VML	3.13	0.76	8	0.564	Fig. 2H	g/min	All groups compared	Two-way ANOVA	Main effect of injury p=0.295; activity p=0.470
		VML Unrestricted		3.21	0.67	12						Interaction injury x activtiy p=0.564
		Naïve Restricted		3.24	0.68	8						
		VML Restricted		3.54	0.69	12						
Q. 12-h Active Carbohydrate Oxidation	Restriction of activity following VML resulted in greatest whole-body carbodhyrate oxidation across 12-hr active period	Naïve Unrestricted	6 weeks post-VML	6.16	1.03	8	**Interaction injury x actvitiy p<0.0001**	Fig. 2H	g/min	All groups compared	Two-way ANOVA	**Main effect of activity p=0.001;** activity p=0.057
		VML Unrestricted		5.47	0.87	12						**Interaction injury x actvitiy p<0.0001**
		Naïve Restricted		5.87	0.94	8						
		VML Restricted		7.88	1.14	12						
R. Glucose Tolerance Testing	Across groups, blood glucose was highest at 30-min post-injection. VML resulted in decreased overall blood glucose across test compared to naive, indicating improved glucose tolerance.	Naïve Unrestricted	0 min	146.1	25.9	8	**Main effect of injury p<0.0001, main effect of time p<0.0001**	Fig. 2I	mg/dl	All groups compared	Three-way ANOVA	**Main effect on injury p<0.0001;** activity p=0.597; **time p<0.0001**
		VML Unrestricted		141.3	23.4	12						Interaction injury x activity x time p=0.246
		Naïve Restricted		148.4	30.7	8						Interaction activity x time p=0.680
		VML Restricted		152.3	45.6	12						Interaction injury x time p=0.403
		Naïve Unrestricted	15 min	360.4	83.9	8						Interaction injury x activity p= 0.191
		VML Unrestricted		346.5	81.2	12						
		Naïve Restricted		335.0	134.5	8						
		VML Restricted		322.3	66.4	12						
		Naïve Unrestricted	30 min	379.5	111.9	8						
		VML Unrestricted		344.7	65.4	12						
		Naïve Restricted		406.5	125.1	8						
		VML Restricted		352.4	102.8	12						
		Naïve Unrestricted	45 min	398.0	92.8	8						
		VML Unrestricted		304.4	62.7	12						
		Naïve Restricted		349.9	128.0	8						
		VML Restricted		322.6	72.3	12						
		Naïve Unrestricted	60 min	300.3	112.7	8						
		VML Unrestricted		304.6	66.4	12						
		Naïve Restricted		358.5	104.5	8						
		VML Restricted		267.7	59.5	12						
		Naïve Unrestricted	120 min	229.1	59.2	8						
		VML Unrestricted		200.3	49.5	12						
		Naïve Restricted		225.5	70.6	8						
		VML Restricted		194.7	41.7	12						
S. Blood Glucose AUC	Regardless of activity, VML resulted in lower blood glucose AUC than injury naive	Naïve Unrestricted	7 weeks post-VML	2464.8	552.1	8	**Main effect of injury p=0.011**	Fig. 2J	A.U.	All groups compared	Two-way ANOA	**Main effect of injury p=0.011;** main effect of activity p=0.826
		VML Unrestricted		2190.3	353.1	12						Interaction injury x activity p=0.462
		Naïve Restricted		2600.7	473.3	8						
		VML Restricted		2116.8	373.9	12						

**4. Histological Evaluation of the left (injured) gastrocnemius muscle**												
Fiber Number	VML resulted in lower overall fiber number in the remaining muscle	Naïve Unrestricted	8 weeks post-VML	8473.50	442.50	4	**Main effect of injury p=0.008**	Fig. 3B	#	All groups compared	Two-way ANOVA	**Main effect of injury p=0.008;** main effect of activity p=0.316
		VML Unrestricted		7057.8	1477.6	4						Interaction injury x activity p=0.978
		Naïve Restricted		8923.50	594.00	4						
		VML Restricted		7532.50	625.70	4						
% NADH+ Fibers-Border	VML resulted in lower % of NADH+ fibers along the border of the gastrocnemius muscle	Naïve Unrestricted	8 weeks post-VML	62.47	6.66	4	**Main effect of injury p=0.043**	Fig. 3C	%	All groups compared	Two-way ANOVA	**Main effect of injury p=0.043;** activity p=0.607
		VML Unrestricted		48.06	10.48	4						Interaction injury x activity p=0.0227
		Naïve Restricted		56.33	6.70	4						
		VML Restricted		50.94	10.52	4						
% NADH+ Fibers-Defect	VML resulted in lower % of NADH+ fibers within the defect of the gastrocnemius muscle	Naïve Unrestricted	8 weeks post-VML	46.36	8.51	4	**Main effect of injury p=0.018**	Fig. 3D	%	All groups compared	Two-way ANOVA	**Main effect of injury p=0.018**; activity p=0.516
		VML Unrestricted		42.36	7.53	4						Interaction injury x activityt p=0.200
		Naïve Restricted		56.07	8.45	4						
		VML Restricted		39.60	5.02	4						
% NADH+ Fibers-Remaining	No differences across groups	Naïve Unrestricted	8 weeks post-VML	66.56	6.79	4	0.259	Fig. 3E	%	All groups compared	Two-way ANOVA	Main effect of injury p=0.883; activity p=0.889
		VML Unrestricted		58.84	1.70	4						Interaction injury x activity p=0.259
		Naïve Restricted		58.88	8.27	4						
		VML Restricted		62.72	13.58	4						
**5. Protein Expression of the proximal portion of the left (injured) gastrocnemius muscle**												
A. Acetyl-CoA Carboxylase (ACC) - muscle	No differences across groups	Naïve Unrestricted	8 weeks post-VML	1.00	0.80	8	0.395	Fig. 4C	A.U.	All groups compared	Two-way ANOVA	Main effect of injury p=0.766; activity p=0.885
		VML Unrestricted		1.34	0.70	4						Interaction injury x activity p=0.395
		Naïve Restricted		1.19	0.97	8						
		VML Restricted		0.95	0.66	4						
B. Phosphorylated Acetyl-CoA Carboxylase (pACC) - muscle	No differences across groups	Naïve Unrestricted		1.00	0.49	8	0.730	Fig. 4D	A.U.	All groups compared	Two-way ANOVA	Main effect of injury p=0.216; activity p=0.705
		VML Unrestricted		0.99	0.26	4						Interaction injury x activity p=0.730
		Naïve Restricted		1.39	1.10	8						
		VML Restricted		1.22	0.39	4						
C. pACC/ACC - muscle	The pACC/ACC ratio was higher with physical activity following VML injury, indicating inactivation of ACC and thus lower fatty acid synthesis.	Naïve Unrestricted		1.00	0.44	8	**Interaction injury x activity p=0.050**	Fig. 4E	A.U.	All groups compared	Two-way ANOVA	Main effect of injury p=0.031; activity p=0.885
		VML Unrestricted		0.64	0.24	4						**Interaction injury x activity p=0.046**
		Naïve Restricted		1.04	0.07	8						
		VML Restricted		1.44	0.68	4						
D. Fatty Acid Synthase (FAS) - liver	No differences across groups	Naïve Unrestricted		1.00	0.33	8	0.117	Fig. 4F	A.U.	All groups compared	Two-way ANOVA	Main effect of injury p=0.528; activity p=0.473
		VML Unrestricted		0.77	0.19	4						Interaction injury x activity p=0.117
		Naïve Restricted		0.76	0.15	8						
		VML Restricted		0.86	0.15	4						
E. GLUT4 - muscle	No differences across groups	Naïve Unrestricted		1.00	0.29	8	0.937	Fig. 4G	A.U.	All groups compared	Two-way ANOVA	Main effect of injury p=0.564; activity p=0.612
		VML Unrestricted		1.12	0.68	4						Interaction injury x activity p=0.937
		Naïve Restricted		1.10	0.51	8						
		VML Restricted		1.25	0.69	4						
F. Fatty Acid Binding Protein 4 (FABP4) - muscle	No differences across groups	Naïve Unrestricted		1.00	0.33	8	0.715	Fig. 4H	A.U.	All groups compared	Two-way ANOVA	Main effect of injury p=0.668; activity p=0.723
		VML Unrestricted		0.77	0.19	4						Interaction injury x activity p=0.715
		Naïve Restricted		0.76	0.15	8						
		VML Restricted		0.86	0.15	4						
**6. Metabolic enzymatic activity of the left (injured) gastrocnemius muscle**												
A. Adiponectin - muscle	Adiponectin concentration in the remaining muscle was higher following VML injury	Naïve Unrestricted	8 weeks post-VML	49.05	7.26	8	**Main effect of injury p<0.0001**	Fig. 6A	ng/ml	All groups compared	Two-way ANOVA	**Main effect of injury p<0.0001;** activity p=0.208
		VML Unrestricted		71.64	9.99	4						Interaction injury x activity p=0.117
		Naïve Restricted		48.00	6.58	8						
		VML Restricted		80.75	4.22	4						
B. Leptin - serum	No differences across groups	Naïve Unrestricted		3742.17	3664.17	8	0.472	Fig. 6B	pg/ml	All groups compared	Two-way ANOVA	Main effect of injury p=0.188; activity 0.966
		VML Unrestricted		1210.82	821.33	4						Interaction injury x activity p=0.472
		Naïve Restricted		2909.39	2395.14	8						
		VML Restricted		2148.82	2537.44	4						
C. Citrate Synthase	No differences across groups	Naïve Unrestricted		2.92	0.31	8	0.373	Fig. 6C	(µmol/min/g)	All groups compared	Two-way ANOVA	main effect of injury p=0.524; activity 0.241
		VML Unrestricted		3.17	0.31	8						Interaction injury x activity p=0.373
		Naïve Restricted		2.88	0.59	8						
		VML Restricted		2.83	0.49	8						
D. Beta-HAD	VML injury resulted in lower Beta-HAD activity when normalized to CS activity	Naïve Unrestricted		15.18	5.91	8	**Main effect of injury p=0.011**	Fig. 6D	(µmol/min/g)	All groups compared	Two-way ANOVA	Main effect of injury p=0.011; activity p=0.948
		VML Unrestricted		13.09	1.84	8						Interaction injury x activity p=0.808
		Naïve Restricted		13.85	7.26	8						
		VML Restricted		12.85	1.59	8						
